# Osteocalcin in human breast milk over the course of lactation

**DOI:** 10.3389/fendo.2025.1715553

**Published:** 2025-11-26

**Authors:** Olivier Prosperi, Beatrice Hanusch, Sophia Naber, Thomas Lücke, Kathrin Sinningen

**Affiliations:** 1 Research Department of Child Nutrition, University Hospital of Pediatrics and Adolescent Medicine, St. Josef-Hospital, Ruhr University Bochum, Bochum, Germany; 2 Department of Anaesthesiology, Intensive Care and Pain Medicine, Ruhr- University Hospital Bergmannsheil, Bochum, Germany; 3 Department of Anesthesiology and Intensive Care Medicine, University Hospital Bonn, Bonn, Germany; 4 University Hospital of Pediatrics and Adolescent Medicine, St. Josef-Hospital, Ruhr University Bochum, Bochum, Germany

**Keywords:** lactation, human milk, osteocalcin, breastfeeding, bone

## Abstract

**Background:**

Breast milk is the best nutrition for newborns. Its composition is dynamic and adapted to the newborn’s individual needs. A variety of bioactive compounds, including bone-related molecules, have been described in breast milk. Osteocalcin (OCN) is a small non-collagenous, bone-derived protein. In its carboxylated form (cOCN) it plays a crucial role in bone metabolism and calcium homeostasis. The undercarboxylated form of osteocalcin (ucOCN) is suggested to have endocrine functions such as influencing neurotransmitter synthesis and glucose homeostasis. This study aimed to analyze whether the bone-related proteins cOCN and ucOCN are present in breast milk at different time points of the lactation phase and whether the amount is affected by lifestyle factors, such as dietary behavior, physical activity, BMI, age, or number of parities.

**Methods:**

98 mothers, aged 31.8 ± 4.7 years, participated in the study. Breast milk was collected at four points during the breastfeeding period (T1: 1–3 days postpartum (pp); T2: 7 days pp; T3: 14 days pp; T4: 3 months pp). Total protein, ucOCN and cOCN were analyzed by BCA (bicinchoninic acid) assay and enzyme immunoassay, respectively. Dietary habits, physical activity, and BMI were assessed.

**Results:**

The median concentration of cOCN in relation to the total amount of protein decreased only marginally during the breastfeeding period (T1: 0.068 [0.021-0.207] ng/mg protein; T4: 0.045 [0.026-0.196] ng/mg protein), with total protein concentrations in breast milk decreasing. In contrast, the relative ucOCN concentration in relation to the total protein concentration was twice as high in the colostrum (0.125 [0.043-0.223] ng/mg protein) compared to mature breast milk (0.055 [0.028-0.091] ng/mg protein). The concentration of ucOCN in colostrum from primiparous women (0.138 [0.041 – 0.342] ng/mg protein) tended to be higher than the concentration in colostrum of multiparous women (0.062 [0.034 – 0.151] ng/mg protein, p = 0.094). This also applied for the cOCN concentration. Other lifestyle factors, e.g., physical activity, BMI, or age showed no associations with ucOCN concentrations in breast milk.

**Conclusion:**

This study indicates that ucOCN and cOCN are components of breast milk. The function of both molecules in infants and mothers, still needs to be uncovered.

**Clinical Trial Registration:**

https://www.bfarm.de, identifier DRKS00023072.

## Introduction

Breast milk is the best nutrition for infants as it contains numerous nutrients required for optimal growth (1). The World Health Organization recommends to exclusively breastfeed for the first 6 months of life and to continue breastfeeding along with appropriate complementary feeding (2). Breastfeeding is associated with a reduced risk of infections and allergic diseases (3, 4). It protects from the development of diabetes mellitus type 2 and lowers the odds for obesity (5).

The composition of human milk is dynamic and changes during the lactation phase from colostrum to mature milk. It contains a wide range of nutrients, especially bioactive proteins (6). Furthermore, the composition varies inter- and intra-individually; it changes over the course of the day and can be influenced by maternal lifestyle factors, e.g., dietary habits (7–9). Although industrially produced infant formula has been optimized through constant research in recent years, it cannot yet fully imitate breast milk (10).

Breastfeeding is a major challenge for the maternal organism. Every day, 300 to 400 mg calcium is excreted into breast milk, the main source of which is the mother’s skeleton (11). Lactational bone loss is suggested to be a result of reduced estradiol levels found in lactating women that leads to an increased bone turnover, reflected by elevated bone resorption as well as bone formation markers such as osteocalcin (12, 13).

Human osteocalcin (OCN), also known as bone γ-carboxyglutamic acid-containing protein, is a small, non-collagenous protein consisting of 49 to 50 amino acids, synthesized and secreted by osteoblasts and odontoblasts, which is the most abundant non-collagenous protein in bone tissue (14). OCN plays a crucial role in bone metabolism and calcium homeostasis (15). There are two forms of OCN: the carboxylated OCN (cOCN) and the undercarboxylated OCN (ucOCN). The post-translational carboxylation occurs through a vitamin K-dependent carboxylase, which produces three γ-carboxyglutamic residues at positions 17, 21 and 24 in OCN (16, 17). Within the resorption lacunae of osteoclasts, the pH is low, which leads to the decarboxylation of osteocalcin and its release into circulation (18). ucOCN has been suggested to have endocrine functions *in vivo*, e.g., influencing neurotransmitter synthesis, which might lead to a reduction in anxiety and depression (19) or improving glucose tolerance by stimulating CyclinD1 and insulin expression in β-cells and adiponectin (20, 21). Furthermore, oral application of ucOCN in mice led to substantial increase of ucOCN in serum after 4 weeks with effects on insulin production and glucose utilization, suggesting that ucOCN can be resorbed partially from the intestine (22).

Breast milk contains a variety of bioactive compounds with immune modulating and growth promoting properties (23). These also include bone-related substances, such as osteoprotegerin, receptor activator of nuclear factor κ B ligand (RANKL) or cross-linked N-telopeptide of type I collagen (NTx), which might influence the function of the mammary gland as well as the growth and development of the neonate (24, 25). The aim of this study was to investigate whether another bone-related protein, cOCN and its undercarboxylated compound ucOCN, are also present in breast milk at different time points of the lactation phase.

Additionally, we aimed to identify maternal lifestyle factors with potential to influence the OCN level in breastmilk such as maternal body mass index (BMI), diet, or the number of parities, which have been shown to affect the composition of human breast milk *per se* (26, 27).

## Methods

### Study design and recruitment

Recruitment was undertaken by study personnel between November 2020 and November 2021. During this time, 100 women who were about to give birth or had just given birth were recruited in two large maternity units in Bochum (St. Elisabeth-Hospital) and Dortmund (Klinikum Dortmund), Germany. Two women withdrew consent shortly after participation.

For inclusion, the study subjects had to be at least 18 years old, fluent in German, willing to fully or partially breastfeed for at least 3 months, and own a freezer for short-term milk sample storage. Exclusion criteria were giving birth to a preterm infant (< 2500 g and/or < 37^th^ week of gestation) and any form of diabetes (diabetes mellitus type 1, type 2 or gestational diabetes mellitus). As a reward for their participation, the participants received financial compensation of up to 30 € and an information booklet on child nutrition.

Written informed consent was obtained from all participants. The subjects were free to withdraw consent at any time. The study was performed in line with the principles of the Declaration of Helsinki and registered at the German Clinical Trials Register (DRKS00023072). Approval was granted by the ethics committee of the Ruhr-University of Bochum, Germany (#20-7006.).

### Sample processing and analysis

**Figure 1:**
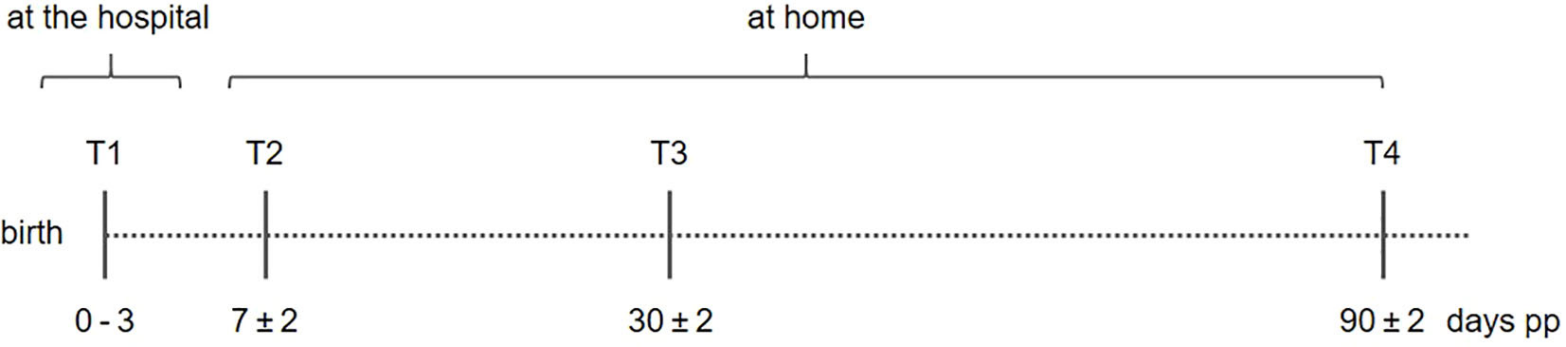
Collection of milk. To ensure the collection of the 3 phases of breastmilk, 4 different time points were selected: T1 (colostrum) at 0–3 days postpartum (pp), T2 (transitory milk) at 7 ± 2 days pp; T3 and T4 (mature breast milk) at 30 ± 2 and 90 ± 2 days pp. At T1 breastmilk was collected at the hospital and the subjects were instructed into usage of the breastmilk pump. At T2 – T4 breastmilk samples were collected at home and immediately stored at -20 °C.

Breast milk samples were collected under standardized conditions at four-time points during the lactation period (**Figure 1**). Milk was collected between 8 am and 12 pm using an electric breast pump (Symphony^®^, Medela, Baar, Switzerland). It was essential that milk was pumped from the same breast for all time points, and mothers were instructed to fully empty the breast in the previous feed. Milk from that breast was pumped entirely, and an aliquot of 5 ml was stored at -18 °C until collection by the study staff. The remaining breast milk was available to feed the infant as needed. To ensure standardized conditions, the staff supported the study participants during the first sample collection. For the subsequent appointments, participants were free to collect the samples either in the presence of the study staff or on their own. If they chose to do it alone, the study staff was available by phone at any time.

The samples were further processed after a maximum storage time of 7 days at -18 °C. Then samples were thawed, vortexed, and centrifuged at 1000 rpm at 4 °C for 10 minutes. Post centrifugation, the liquid phase was removed and transferred into a new tube; the fat phase was discarded, and the process was repeated once again. During this process, samples were kept on ice. The fat-reduced samples were then stored at -80 °C until further analysis.

Total protein was measured using a commercially available kit (BCA (bicinchoninic acid) Protein Assay Kit, Thermo Scientific™ Perice™, Rockfordt, USA). cOCN and ucOCN concentrations were analyzed using commercially available enzyme immunoassay (EIA) kits (#MK111 Gla-OC, #MK118 Glu-OC; Takara Bio Europe, France).

### Data collection

To compare the measured cOCN and ucOCN concentrations, both values were adjusted with the total protein concentration.

The mother’s habitual diet, physical activity and health status were recorded at T1 and T4 using questionnaires from *German Health Interview and Examination Survey for Adults* (DEGS) (28). Questionnaires also included questions regarding the mother’s age and school education. Mothers could choose between degrees *Hauptschulabschluss*, *Realschulabschluss* and *Abitur*, which corresponds to basic, secondary, and higher secondary education. Dietary habits were evaluated at T1 and T4 using the Healthy-Eating-Index (HEI), which is used to assess and quantify dietary quality. It compares the intake of 15 food groups (drinks, vegetables, fruits, fish, cereals, side dishes (potatoes, pasta, rice), nuts, milk and dairy products, cheese, eggs, meats and sausages, fat, sweets and snacks, sugary drinks and alcohol) with the German Nutrition Society (DGE) recommendations for a healthy diet (29). For 54 food items, mean consumption frequency (ranging from “never” to “more than 5 times per day”) over the last four weeks and mean portion sizes (e.g., teaspoons, tablespoons, cups, plates) were assessed. The score ranges from 0 (no adherence to recommendations) to 100 (highest adherence to recommendations) points (30). For further analysis, subjects were divided into tertiles according to their achieved HEI. As we expected lower influence of nutrition in participants with a HEI in the second HEI tertile, only the first tertile (= HEI low, HEI < 51.74) was compared to the third tertile (= HEI high, HEI > 58.45).

Furthermore, participants answered questions regarding their weight, height and physical activity at each time point. They were asked how often and for how long they had exercised on average per week over the last 4 weeks. Additionally, mothers were also asked how many days a week they were physically active to the point of sweating or getting out of breath and how long (in minutes) they were physically active on these days. Classifications of physical activity were based on the recommendation of the World Health Organization for pregnant and breastfeeding women to engage in at least 150 minutes of moderate-intensity aerobic physical activity per week (31). Participants who engaged in at least 150 minutes of physical activity per week (exercising or sweating during other activities such as walking or housework) were considered physically active, and those who engaged in less than 150 minutes of physical activity per day were considered sedentary.

BMI was calculated from height and weight ((kg)/(height in m)^2^), and subjects with a BMI of ≥ 25 kg/m^2^ were considered overweight according to the WHO (32). Missing BMI values at T1 were supplemented with values from T2, if available.

To determine the influence of age on cOCN and ucOCN concentrations, the cohort was divided into a group < 35 years and a group ≥ 35 years of age, based on the age at which pregnancies are considered to be higher risk (33). Number of previous births was recorded and mothers were divided into primigravida (first pregnancy; miscarriages and abortions not considered) and multigravida (more than one delivery).

### Statistics

SPSS version 29 (IBM Corp., Armonk, NY) was used for data analysis. Data was tested for normal distribution using Shapiro-Wilk test. OCN concentrations of different time points were analyzed using Friedman test for one-way repeated measures analysis of variance by ranks. Correction for multiple testing was performed using Bonferroni. Group differences of non-normally distributed data were analyzed using Mann-Whitney U test. Values of p < 0.05 were considered significant.

## Results

### Maternal characteristics

**Figure 2:**
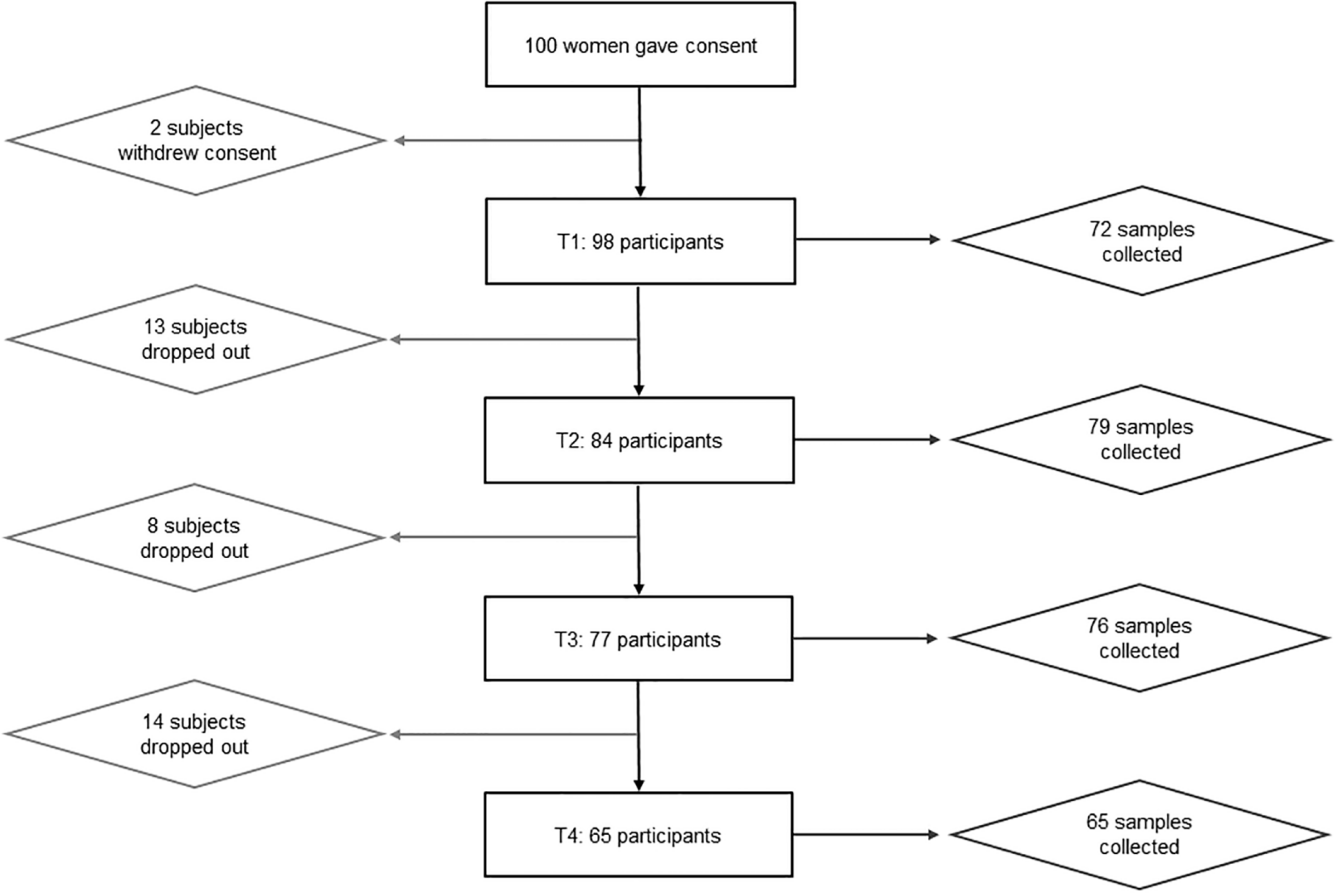
Flowchart showing participation. Out of 100 women that gave consent, only 65 participated until the end. Two participants withdrew their declaration of consent before the first appointment. Reasons for early termination of participation included, e.g., premature weaning, mastitis, discomfort during breastfeeding. At the first time point, only 72 out of 98 subjects were able to provide a breastmilk sample due to issues with breastfeeding initialization or too small sample volumes.

Out of the 100 mothers that were recruited, two withdrew their consent shortly after participation (**Figure 2**). Of the remaining 98 participants, 72 provided a milk sample at T1, 79 at T2, 76 at T3 and 65 at T4 (**Figure 2**). At T1, fewer samples were collected than at T2 due to issues with breastfeeding initialization or small sample volume (**Figure 2**). As 11 mothers were lost to follow up at T4, most comparisons were conducted with T3.

At the time of delivery, the participating mothers were between 21 and 42 years old (31.8 ± 4.7 years, **Table 1**), and for 55% this was their first pregnancy (primigravida). On average, mothers had gained 14.8 ± 5.8 kg during this pregnancy and had a mean BMI of 28.3 ± 6.7 at delivery (T1). 78% of mothers had attended higher secondary education, while 8% had basic education. Mothers achieved a HEI of 55.2 ± 9.3 at T1 and 52.3 ± 8.9 at T4 (**Table 1**).

**Table 1:** Maternal characteristics.

Parameter	n	Mean ± SD	Range (min-max)
Age, years	71	31.8 ± 4.70	21-42
BMI at T1, kg/m^2^	73	28.3 ± 6.73	20.3-58.8
BMI at T3, kg/m^2^	69	26.2 ± 5.13	16.9-50.0
Weight gain during pregnancy, kg	69	14.8 ± 5.80	5.0-42.0
Physical activity at T1, min/wk	85	133 ± 150	0-660
Physical activity at T3, min/wk	85	71.1 ± 118	0-510
HEI at T1	60	55.2 ± 9.28	36.2-79.5
HEI at T4	54	52.3 ± 8.85	32.2-75.3
Primigravida, n (%)	73	43.0 (55.1)	NA
School education, n (%)^#^ Higher secondary Secondary Basic	78	61 (78.2) 11 (14.1) 6 (7.69)	NA

BMI body mass index; T1: 1–3 days postpartum (pp), T3: 30 ± 2 days pp, T4: 90 ± 2 days pp, HEI, Healthy-Eating-Index; NA, not applicable; wk, week; ^#^ categories of school education corresponds to the degrees *Hauptschulabschluss*, *Realschulabschluss* and *Abitur* from the German school system.

### OCN in breast milk

The concentration of cOCN in relation to the total protein concentration remained stable during the breastfeeding phase (**Table 2**), while ucOCN was almost twice as high (0.125 [0.043-0.223] ng/mg protein) in colostrum compared to the subsequent breastfeeding phases (p < 0.001 for all time points, **Table 2**). Similarly, the ucOCN/cOCN ratio was highest in colostrum and decreased during the breastfeeding phase (**Table 2**). Concentrations of total protein as well as protein-corrected cOCN and ucOCN from all participants without complete sample sets at all time points are displayed in **Supplementary Table S1**.

**Table 2:** Concentrations of total protein, carboxylated (cOCN) and undercarboxylated osteocalcin (ucOCN) over the course of lactation in mothers with complete sample sets.

Parameter	T1	T2	T3	T4	p
Protein (mg/ml) n = 46	19.48 [13.66-28.45]	15.96 [9.264-19.20]	11.09 [8.305-13.78]	10.39 [5.851-12.66]	< 0.001^a^
ucOCN (ng/mg protein) n = 43	0.125 [0.043-0.223]	0.070 [0.032-0.106]	0.045 [0.028-0.073]	0.055 [0.028-0.091]	< 0.001^b^
cOCN (ng/mg protein) n = 34	0.068 [0.021-0.207]	0.037 [0.025-0.143]	0.047 [0.031-0.121]	0.045 [0.026-0.196]	0.662
ucOCN/cOCN n = 34	1.686 [0.904-3.964]	1.052 [0.529-2.242]	0.899 [0.553-1.416]	0.924 [0.522-1.348]	< 0.001^c^

T1: 1–3 days postpartum (pp), T2: 7 ± 2 days pp, T3 30 ± 2 days pp, T4: 90 ± 2 days pp.

Subgroup analyses: ^a^ T 1 vs. T2: p = 0.007, T1 vs. T3: p < 0.001, T1 vs. T4: p < 0.001, T2 vs. T3: p = 0.001, T2 vs. T4: p < 0.001; ^b^ T1 vs. T2: p < 0.001, T1 vs. T3: p = < 0.001, T1 vs. T4: p < 0.001, T2 vs. T3: p = 0.021; ^c^ T1 vs. T2: p = 0.002, T1 vs. T3: p < 0.001, T1 vs. T4: p < 0.001; median [25th-75th percentile]; Friedman-Test, Bonferroni-corrected for multiple testing.

### Lifestyle factors and OCN in breast milk

At both time points (T1 and T3), the majority of study participants were physically active for less than 150 minutes per week, especially 30 days pp (71.1 ± 118 min; **Table 1**). ucOCN and cOCN concentrations in breast milk were not significantly different between physically active and sedentary mothers (**Table 3**, **Supplementary Table S2**). The same applied to participants with high HEI and low HEI.

**Table 3:** Concentrations of undercarboxylated (ucOCN) and carboxylated osteocalcin depending on selected lifestyle factors in colostrum (1-3 days postpartum).

ucOCN (ng/mg)			cOCN (ng/mg)		
Age < 35 years	Age ≥ 35 years	*p*	Age < 35 years	Age ≥ 35 years	*p*
n = 39	n = 24		n = 34	n = 22	
0.125	0.095		0.049	0.038	
[0.040-0.215]	[0.039-0.278]	0.777	[0.020-0.157]	[0.019-0.149]	0.814
BMI < 25	BMI ≥ 25		BMI < 25	BMI ≥ 25	
n = 20	n = 40		n = 17	n = 36	
0.116 [0.034-0.376]	0.116 [0.034-0.376]	0.456	0.064 [0.020-0.322]	0.037 [0.019-0.135]	0.424
Physically active	Sedentary		Physically active	Sedentary	
n = 28	n = 42		n = 21	n = 41	
0.127 [0.041-0.278]	0.109 [0.040-0.214]	0.801	0.043 [0.019-0.138]	0.054 [0.019-0.172]	0.749
Primigravida	Multigravida		Primigravida	Multigravida	
n = 35	n = 26		n = 30	n = 25	
0.138 [0.041-0.342]	0.062 [0.034-0.151]	0.094	0.059 [0.021-0.201]	0.029 [0.013-0.124]	0.082
HEI low	HEI high		HEI low	HEI high	
n = 17	n = 18		n = 13	n = 17	
0.138 [0.036-0.376]	0.091 [0.035-0.180]	0.373	0.063 [0.014-0.351]	0.065 [0.020-0.282]	0.851

ucOCN and cOCN concentrations were comparable between mothers with higher-risk pregnancy (≥ 35 years of age) and mothers < 35 years of age (**Table 3**, **Supplementary Table S2**). Furthermore, ucOCN and cOCN concentrations did not show any significant differences when comparing the BMI > 25kg/m^2^ group to the BMI ≤ 25kg/m^2^, neither at T1 nor at T3.

At T1, the protein concentrations of ucOCN and cOCN were slightly but insignificantly lower in multiparous women (ucOCN: 0.062 [0.034 – 0.151] ng/mg protein; cOCN: 0.029 [0.013 – 0.124] ng/mg protein) compared to primiparous women (ucOCN: 0.138 [0.041 – 0.342] ng/mg protein; cOCN: 0.059 [0.021 – 0.201] ng/mg protein). At T3 ucOCN tended to be lower in multiparous mothers (0.04 [0.02 – 0.06] ng/mg protein) than in primiparous (0.05 [0.03 – 0.10] ng/mg protein), but this difference also lacked significance (p = 0.056). At T3, this trend was no longer seen in cOCN (**Supplementary Table S2**).

## Discussion

This study analyzed cOCN and ucOCN concentrations in human breast milk during the first 4 months of the breastfeeding period. While ucOCN was highest in colostrum and decreased in the following months, cOCN decreased only marginally over the course of lactation.

In 1993, Pittard et al. were the first to analyze total OCN in transitory breast milk of 11 women via radioimmunoassay method. However, total OCN concentrations were below 0.1 ng/ml breast milk (34), while we determined 0.094 – 13.02 ng/ml ucOCN and 0.057 – 10.09 ng/ml cOCN (both not adjusted for protein) in transitory breast milk (T2: 7 ± 2 days pp). An explanation could be differences in handling the milk samples. While Pittard et al. centrifuged once and discarded the cream, we first vortexed the samples and additionally centrifuged twice with discarding the cream after each centrifugation (34). Vortexing the samples might have led to dissolving proteins packed into milk fat globules or cells leading to higher concentrations of ucOCN and cOCN. Additionally, the double centrifugation might have led to smaller interference of fats in the assay and thereby granted higher concentrations of the OCN proteins.

To our knowledge the only previous measurement of OCN in breast milk was done by Pittard et al. (34). Therefore, the following discussion mainly focuses on observed changes in serum OCN. We cannot rule out, that breast tissue actively secretes OCN. To answer this question, it would therefore be useful to conduct follow-up studies comparing OCN concentrations in the blood with those in breast milk of breastfeeding mothers.

Transiently higher ucOCN concentrations in colostrum could have multiple causes: acute stress (35) caused by childbirth, physical activity of active labor (36), and potential mimicry of placental ucOCN secretion (37) might only be a few possible explanations. With regard to the latter point placenta was found to actively secrete ucOCN, which is resorbed by the fetus (37). ucOCN secretion into colostrum might therefore mimic this active secretion to provide the newborn with ucOCN with unknown effects for the child.

Acute stress response was found to be mediated by ucOCN in mice and humans (35). This effect was observed to be facilitated via glutamatergic neurons in bone, inhibiting γ-carboxylase in osteoblasts. Thereby, ucOCN is actively secreted by osteoblasts without changes in osteoclast activity (35). The resulting acute stress response is mediated by ucOCN inhibiting parasympathetic tonus (35). Potentially, higher ucOCN concentration in colostrum might result from the acute stress response during birth.

Interestingly, lower OCN blood concentration was observed in patients 48h after elective abdominal surgery, myocardial infarction or fracture (38). As the birth of a child potentially has a similar stressful effect on the body, the increase in OCN in breast milk is particularly interesting, especially in case of cesarean section. As Dockree et al. did not observe elevated blood lactate after cesarean section, but after vaginal delivery, birth mode potentially might be relevant (39). High intensity interval exercise not only leads to elevation of lactate but also to an elevated ucOCN blood concentration in men and women, while cOCN was unchanged (36). Additionally, in ovariectomized mice, lactate injections as well as high intensity interval training saved bone loss in both groups by stimulating osteoblast activity (40). Like physical activity, vaginal birth also leads to elevated blood lactate concentrations, possibly as a result of active pushing during the second half of active labor (39). Transient elevation of ucOCN might therefore be a result of active labor. Regrettably, mode of birth was not documented; therefore, we cannot exclude a potential effect of cesarean section vs. spontaneous birth on OCN in colostrum. This would also be of interest in consideration of lower OCN blood concentration after abdominal surgery (38). As ucOCN ranges in breast milk were quite large, elevation from active labor and decrease due to cesarean section might both occur, increasing the observed range of OCN in breast milk. Future research into the effects of birth mode on OCN breast milk could be of interest.

As to our knowledge, no OCN expressing cells have been described in human breast tissue, we expect the OCN observed in breast milk to originate from bone. Therefore, varying OCN concentrations could be indicative for differences in bone turnover. Bone turnover was found to be elevated in lactating women and mice, including significantly higher serum levels of OCN in lactating women than in non-lactating women (12, 41, 42). The regulation of bone turnover during lactation is primarily influenced by estradiol suppression due to prolactin increase and gonadotropic-releasing hormone which are both triggered by suckling (43). It would be of great interest to investigate whether the concentration of OCN in breast milk correlates with the extent of bone loss.

We found high fluctuations in OCN between individual mothers, particularly in the colostrum. However, association with different lifestyles factors such as physical activity, habitual diet, and BMI were not detected. Only parity might influence OCN concentration as we found a trend for higher OCN in primiparous mothers.

Previous research found a similar effect of the number of parities on the concentration of immune factors and lactocytes in breast milk (26, 27, 44). Akhter et al. discussed that primiparous women have higher total protein concentrations in serum than multiparous women because primiparous women are, on average, younger than multiparous women (26). However, we were unable to confirm this effect of age when examining the concentration differences in relation to age. Additionally, we adjusted OCN concentration for the total protein concentration in breast milk. Effects of higher total protein concentration in primiparous mothers would have been nullified by this method. We still observed a trend towards higher OCN concentration in breastmilk of primiparous mothers. Tomaszewska et al. discussed anatomical changes in the breast tissue as well as changes in breast milk microbiome of multiparous mothers (44). As we did not take tissue samples or characterize breast milk microbiome, these areas might also influence OCN concentration. Further research into breast tissue changes or influence of microbiome on OCN might identify more unknown sources of this protein.

Another explanation for the reduced OCN concentrations in breast milk of multiparous women could be a lower stress level compared to primiparous women (45, 46). As stress is associated with changes in OCN in healthy adults, patients with diabetes type 2 and depression as well as in mice (19, 35, 47), differences in stress level between primiparous and multiparous women might influence OCN in breast milk. Indeed, Chinese women perceived lower stress levels during their second pregnancy than during their first pregnancy (45), especially when the subjects had positive childbirth experiences (46). The extent to which the mothers felt stressed during pregnancy was not surveyed in our questionnaire. The connection between increased OCN concentrations due to stress requires further studies. Therefore, chronic stress and acute stress both might influence OCN concentration in breast milk.

In comparison to findings from other studies showing that an elevated BMI correlates with ucOCN concentrations (48), we could not find any association with BMI. As pregnancy is associated with physiological weight gain and weight loss was observed between T1 and T3, relevance of BMI on OCN in breastmilk might be debatable. BMI prior to and weight-gain during pregnancy is associated with several health outcomes for mother and child, but measurement of overweight at the end of pregnancy might not be useful as a parameter (49–51). In particular, using a BMI > 25 kg/m^2^ as threshold for overweight, might not be an appropriate indicator of overweight shortly after giving birth. Longitudinal analysis of women prior to pregnancy could help identifying the influence of weight gain during pregnancy on OCN in mother’s serum as well as breast milk.

The key question is whether OCN in breast milk has any significance for the health and development of the child. ucOCN in particular is thought to have hormonal effects and, as already mentioned, is associated with a lower risk of diabetes or cardiovascular disease (52). However, it is questionable whether the protein is absorbed by the child’s organism. Experimental data suggests that orally ingested OCN survives the gastrointestinal passage and is absorbed in the small intestine of mice (22). Additionally, a recent study described significantly higher median OCN serum concentrations in 4-month-old breastfed children compared to formula-fed children (53). However, whether OCN actually comes from breast milk or stems from an increased endogenous synthesis in breastfed infants still needs to be clarified.

### Limitations

The study’s major weakness is that the study population is quite small, especially for subanalyses. Additionally, mass spectrometry potentially would have been the more accurate method to identify OCN, although this method is not established for OCN in breast milk either (54). Only one study previously analyzed OCN in breastmilk. As Pittard et al. used RIA, we chose a similar method even though the manual not explicitly stated breastmilk as a suitable medium (34). Thawing twice, compared to only once in Pittard et al., might additionally have led to smaller concentration of OCN due to breakdown of the protein (34). Keeping this in mind, we still were able to detect higher OCN values than Pittard et al. (34). Further studies on detection of OCN in breastmilk are needed to optimize measurement of OCN in general, and especially in breastmilk.

The main part of the discussion focuses on changes in OCN in serum which might translate into changes in breast milk, since only one previous study analyzed OCN in breast milk (34). Unfortunately, blood was not collected from participating mothers; therefore, we cannot analyze further parameters of maternal bone turnovers. As previously mentioned, birth mode might have influenced ucOCN concentration in colostrum. Including this information could have helped identify causes for the high variance of measured concentrations. It is unclear whether birth duration could also have affected the observed results in colostrum. Further studies should therefore include both characteristics.

As pregnant women are advised to supplement folic acid and iodine, many women tend to consume multivitamins marketed for pregnancy (55, 56). Here vitamin D and vitamin K might have been included. Both of these vitamins could have had an influence on OCN serum concentrations, but the validated questionnaire we used for information on habitual diet did not include questions on supplementation. Finally, self-reported data on habitual diet and physical activity always entail possible bias due to reporting errors, even though habitual diet was comparable to those of female adults in the general population (29).

## Conclusion

The results of this study indicate that ucOCN and cOCN are components of breast milk, the concentration of which decrease from colostrum to mature breast milk during the lactation period. Nevertheless, further research is needed to confirm these results and to decipher what function ucOCN and cOCN might have for the newborn.

## Data Availability

The raw data supporting the conclusions of this article will be made available by the authors, without undue reservation.
