# Supplementary material for: Osteocalcin in human breast milk over the course of lactation
Source: Front Endocrinol (Lausanne). 2025 Nov 26;16:1715553. doi: 10.3389/fendo.2025.1715553 (PMC12689333; doi:10.3389/fendo.2025.1715553)
Supplement: Supplementary file 1 [file Table1.docx]

**Table S1.** Concentrations of total protein and carboxylated (cOCN) and undercarboxylated osteocalcin (ucOCN) over the course of lactation in all mothers

|  | **T1** | **T2** | **T3** | **T4** |
| --- | --- | --- | --- | --- |
| **Protein (mg/ml)** | n = 71 | n = 78 | n = 76 | n = 65 |
|  | 19.28  [14.44-28.98] | 15.96  [9.294-19.49] | 11.87  [8.439-14.91] | 10.37  [7.020-12.55] |
| **ucOCN (ng/mg protein)** | n = 70 | n = 75 | n = 73 | n = 63 |
|  | 0.116  [0.040-0.227] | 0.063  [0.029-0.097] | 0.046  [0.028-0.083] | 0.051  [0.025-0.091] |
| **cOCN (ng/mg protein)** | n = 62 | n = 65 | n = 67 | n = 61 |
|  | 0.043  [0.019-0.157] | 0.038  [0.024-0.129] | 0.042  [0.031-0.076] | 0.038  [0.026-0.139] |
| **ucOCN/cOCN** | n = 61 | n = 63 | n = 64 | n = 62 |
|  | 1.672  [1.010-3.983] | 1.034  [0.532-2.074] | 0.899  [0.595-1.372] | 0.811  [0.533-1.413] |

T1: 1-3 days postpartum (pp), T2: 7 ± 2 days pp, T3: 30 ± 2 days pp, T4: 90 ± 2 days pp. median [25th-75th percentile]

|  | **ucOCN (ng/mg)**  **Table S2.** Concentrations of undercarboxylated (ucOCN) and carboxylated osteocalcin in mature milk depending on selected lifestyle factors | | | **cOCN (ng/mg)** | | |
| --- | --- | --- | --- | --- | --- | --- |
|  | **Age < 35 years** | **Age ≥ 35 years** | ***p*** | **Age < 35 years** | **Age ≥ 35 years** | ***p*** |
| **T3** | n = 41 | n = 27 |  | n = 39 | n = 22 |  |
|  | 0.046 | 0.048 |  | 0.037 | 0.049 |  |
|  | [0.027-0.093] | [0.026-0.073] | 0.817 | [0.021-0.076] | [0.034-0.093] | 0.176 |
|  | **BMI < 25** | **BMI ≥ 25** |  | **BMI < 25** | **BMI ≥ 25** |  |
| **T3** | n = 30 | n = 34 |  | n = 23 | n = 34 |  |
|  | 0.037  [0.025-0.055] | 0.052  [0.030-0.055] | 0.150 | 0.039  [0.030-0.089] | 0.041  [0.029-0.068] | 0.795 |
|  | **Physically active** | **Sedentary** |  | **Physically active** | **Sedentary** |  |
| **T3** | n = 13 | n = 60 |  | n = 12 | n = 55 |  |
|  | 0.050  [0.027-0.082] | 0.046  [0.028-0.084] | 0.773 | 0.055  [0.032-0.190] | 0.039  [0.030-0.072] | 0.360 |
|  | **Primigravida** | **Multigravida** |  | **Primigravida** | **Multigravida** |  |
| **T3** | n = 37 | n = 28 |  | n = 34 | n = 25 |  |
|  | 0.053  [0.028-0.104] | 0.040  [0.021-0.063] | 0.056 | 0.051  [0.028-0.117] | 0.037  [0.032-0.050] | 0.304 |
|  | **HEI low** | **HEI high** |  | **HEI low** | **HEI high** |  |
| **T4** | n = 16 | n = 17 |  | n = 15 | n = 17 |  |
|  | 0.040  [0.028-0.092] | 0.055  [0.025-0.087] | 0.885 | 0.057  [0.028-0.129] | 0.043  [0.029-0.108] | 0.692 |

BMI, body mass index; cOCN, carboxylated osteocalcin; HEI, healthy eating index; HEI low, 1^st^ tertile (HEI < 49.33); HEI high, 3^rd^ tertile (HEI > 56.61); physically active: at least 150 minutes of physical activity per week ; sedenctary: < 150 minutes of physical activity per week; ucOCN, undercarboxylated osteocalcin; T3: 30 ± 2 days pp; T4: 90 ± 2 days pp median [25th-75th percentile]; Mann-Whitney U test
